# Soil bacterial community structure of mixed bamboo and broad-leaved forest based on tree crown width ratio

**DOI:** 10.1038/s41598-020-63547-x

**Published:** 2020-04-16

**Authors:** Mei-man Zhang, Shao-hui Fan, Feng-ying Guan, Xin-rong Yan, Zi-xu Yin

**Affiliations:** State key lab for Bamboo and Rattan Science, International Centre for Bamboo and Rattan, Beijing, 100102 China

**Keywords:** Forest ecology, Forestry

## Abstract

Moso bamboo (*Phyllostachysheterocycla* (Carr.) Mitford cv. *Pubescens*) is an economically valuable plant in bamboo production areas of southern China, for which the management mode is crucial for improving the comprehensive benefits of bamboo forest stands. In this respect, mixed forested areas of bamboo and broad-leaved tree species can provide sound ecological management of bamboo in forestry operations. To further this goal, an outstanding question is to better understand the spatial distribution of soil bacterial communities in relation to the proportion of mixed in bamboo and broad-leaved forest. We analyzed soil bacterial community diversity and composition along a proportional gradient of 0–40% mixed-ratio (as represented by the width and size of the broad-leaved tree crown over the plot area) of bamboo and broad-leaved forest in Tianbao Yan Nature Reserve using the highthroughputsequencing of the 16S rRNA gene.Specifically, the sampling plots for the mixed proportions were divided according to the percentage of summed projected area of live broadleaf tree crowns. The main broad-leaved species in the five mixed ratio plots are the same. Each plot was 20 m × 20 m in size, and a total of 15 plots were established, three per forest ratio class. From each plot, soil samples were taken at the surface (0–10 cm depth) in December 2017. Our analysis revealed that soil bacterial diversity community structure and dominant flora changed under different mixing ratios of bamboo and broad-leaved trees. In the stand with a mixed ratio of 10–20%, the bacterial diversity index is higher; however, the diversity was lowest in the 20–30% stands. Among the 20–30% forest soil, *Acidobacteria* (*Solibacteria*, *Solibacteriales*, *Acidobacteriales*) was more abundant than in soils from other mixed-ratio stands.Redundancy analysis showed that mixed forest stand structure, soil pH, organic carbon, total nitrogen, and soil moisture all contributed to shaping the bacterial community structure. Changes in microbial communities were associated with species diversity in tree layers, availability of soil nutrients (SOC and TN), and changes in soil physical properties (MS, pH). Together, these empirical results suggest that different mixing ratios in the bamboo–broad-leaved mixed forest could influence the soil bacterial community structure indirectly, specifically by affecting the soil physical and chemical properties of the forest.

## Introduction

Soil microbes are the driving force behind ecosystem material transformation and nutrient cycling, and they are widely involved in the cyclical transformation of soil organic matter and inorganic matter^1–4^. Microbial diversity is an important way to gauge soil fertility, and it can serve as a sensitive indicator for predicting soil quality changes and processes, such as soil chemical cycles, detoxification of pollutants, formation of soil structure, and decomposition of organic matter^1^. Furthermore, it is well known that vegetation–soil environmental interactions can change the spatial distribution characteristics of soil-dwelling microorganisms^4^. The distribution pattern and functional characteristics of soil microorganisms may have important effects on the aboveground growth of plants^5,6^. Conversely, the type, quantity, and composition of vegetation can alter the structure of the soil microbial community and its diversity^7,8^, such that both the composition and structure of a soil microbial community is affected by the differing forest management practices. For example, to maintain and increase bamboo yield in bamboo production forests, common management measures include the regular removal of underlying vegetation, in addition to farming and fertilization^9–12^.

Moso bamboo forest has a well-developed underground root system. China has approximately 30% of the world’s bamboo resources that, according to incomplete statistics,encompass an area of 6.16 million hectares^13,14^, with more than 70% of these bamboo forests consisting of Moso bamboo (*Phyllostachysheterocycla* (Carr.) Mitford cv. *Pubescens*) is characterized by its palatable shoots and versatile culms^15^. Not surprisingly, moso bamboo forest has become an important forestry and economic plant in the bamboo production areas of southern China because of its high growth rate, short rotation time, high productivity, earlymaturation, and its many commercial applications^16–18^.

Mixing broad-leaved forest tree species with moso bamboo individuals is now a common management practice in China. A long period of artificial operations and farmer’s pursuit of economic gains are thought to have contributed to bamboo–broad-leaved forest reduction in Fujian Province, where moso bamboo is the main forestry resource. Conventional moso bamboo forest management was unreasonable, in that inappropriate management diminishes bamboo forest soil quality. Numerous studies have shown that appropriate mixing with other broad-leaved tree species in the management of bamboo can increase overall forest diversity and change the vegetation type, while also potentially improving the stability and productivity of the ecosystem^19–23^. By contrast, in pure bamboo forest, the plant diversity is generally low, and bamboo can release allelochemicals from their leaves whose allelopathic effects reduce the abundance and species richness of seeds under the bamboo canopy, which can lead to changes in plant community composition and species diversity^9^. Long-term management of pure bamboo forest stands results in lower long-term productivity and low soil quality^24,25^. Several studies that investigated different mixing ratios of bamboo and broad-leaved mixed forests have focused on stand spacing, stand biomass, and their ecological benefits. For example, by addressing the spatial competition of bamboo and broad-leaved tree under different mixing ratio conditions^26^, the impact of the mixing ratio on the aboveground biomass of forests^15^ and changes in local hydrology were studied^27^. However, few reports have examined theimpact of proportion in mixed broad-leaved forest trees with moso bamboo, especially in terms of their crown width ratios, on the structure and diversity of the bacterial community of these forest soils.

Thus, this study was conducted in five different mixed ratio bamboo and broad-leaved forests in the Tianbaoyan Nature Reserve to evaluate: (1) the effects of broad-leaved forest ratios on plant communities and soil physicochemical characteristics; and (2) the impact of changing broad-leaved forest ratios on soil microbial community structure and diversity. The highthroughputsequencing of the 16 S rRNA gene was used to systematically analyze the local soil bacterial communities, and the relationships between the proportion of broad-leaved forest trees and microbial community structure, diversity in bamboo and broad-leaved mixed forests were analyzed. This study provides insight into community structure and diversity of soil bacteria under varying broadleaf tree proportions. The results of this study provide a vital theoretical basis for bamboo forest management inTianbao Yan Nature Reserve, China. We also hypothesized that microbial community structure and diversity of soil in bamboo and broad-leaved mixed forests differed according to the latter’s mixing ratio. When the mixing ratio is different, the corresponding forest micro-environment will markedly diverge, generating dissimilar vegetation types and soil physical and chemical properties, which, in turn, indirectly shape the respective forest soil’s microbial community structure.

## Results

### Plant communities and soil physicochemical properties

The SR1 value of the A1 site was lowestamong the five bamboo and broad-leaved mixed forest sites, whereas it was the highest for C1 (5). Overall, the SR1 increased as the mixing ratio went from A1(1) to C1 (5), and it decreased from C1 (5) to E1(4) (Table 1). In addition, the SR2 increased as the mixing ratio went from A1(1) to E1 (4), while the soils of C1 and E1 forest stands had lower pH and moisture content (Table 1). The SOC increased as the mixing ratio went from A1(32) to C1(39.85), and decreased as the mixing ratio went from C1(39.85) to E1(37.94), with B1(22.13) being the lowest. In general, both TN and TP in C1 had higher values; the lowest values were in D1 forest stands. Furthermore, the C:N ratios were comparable among the five bamboo and broad-leaved mixed forest sites (6.05–10.84), although D1 and E1 had higher values (10.84 and 9.82, respectively).

**Table 1:** Plant communitiesand soil physicochemical characteristics of the five sampled sites of mixed bamboo–broad-leaved forest.

Forest type	SR1	SR2	pH	MS (%)	SOC (g/kg)	TN (mg/g)	TP (mg/g)	C/N
A1	2	1	4.80 ± 0.04ab	15.6 ± 0.9b	32 ± 14a	4.8 ± 0.4a	0.10 ± 0.01a	6.3 ± 2.6a
B1	3	1	4.83 ± 0.05ab	15.0 ± 1.2b	22 ± 1a	3.7 ± 0.4bc	0.097 ± 0.006a	6.1 ± 0.8a
C1	5	3	4.71 ± 0.03b	23.3 ± 3.7a	40 ± 6a	4.5 ± 0.2ab	0.11 ± 0.02a	8.8 ± 0.9a
D1	4	4	4.86 ± 0.04a	14.5 ± 2.2b	39 ± 12a	3.5 ± 0.3c	0.09 ± 0.02a	10.8 ± 1.2a
E1	4	4	4.72 ± 0.04b	28.9 ± 3.2a	38 ± 10a	3.8 ± 0.2abc	0.090 ± 0.006a	9.8 ± 1.1a

SR1, tree layer species richness; SR2, update layer species richness;MS, soil moisture; SOC, soil organic carbon; TN, soil total nitrogen; TP, soil total phosphorus; C/N, carbon nitrogen ratio.Relabel different mixing ratios of bamboo and broad-leaved mixed forest with A-E (10% or less(A1), 10–20%(B1), 20–30%(C1), 30–40%(D1), and more than 40%(E1). Values are means ± standard error (n = 3). Different lowercase letters indicate significant at level of 0.05.

**Figure 1:**
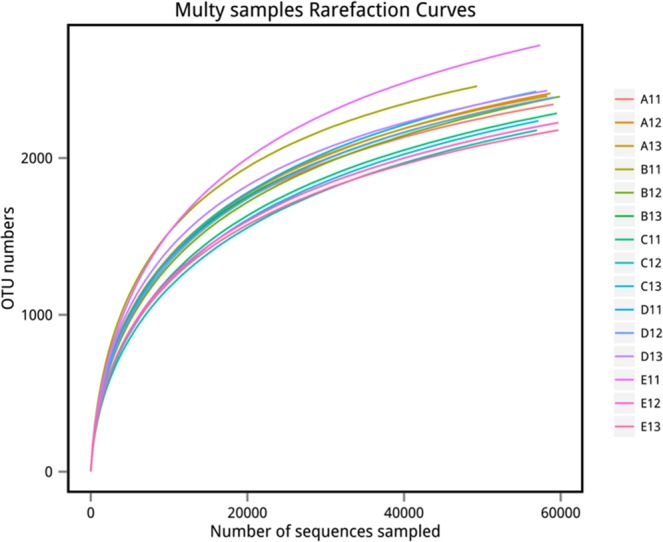
Rarefaction curves of 16S rDNA for high-throughput sequencing of 15 soil bacteria communities.

**Figure 2:**
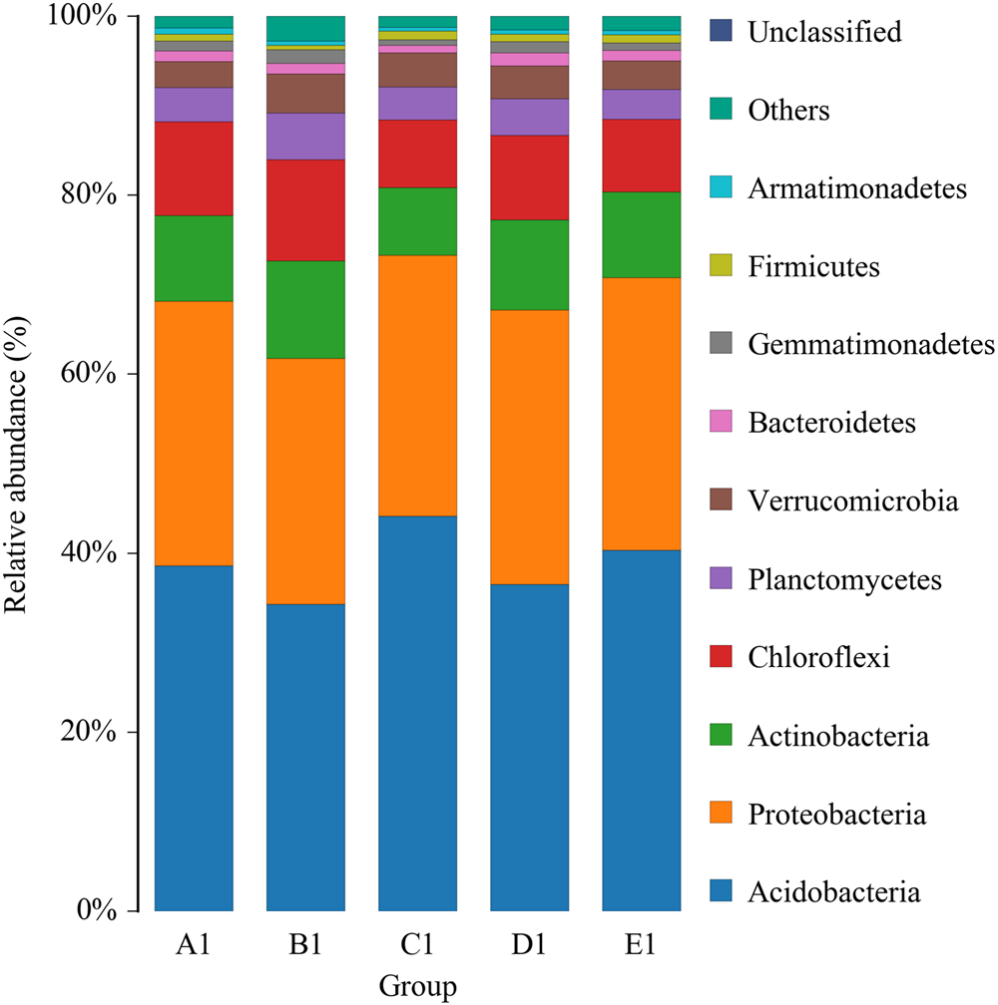
The community composition ofdominant bacteria phyla along mixing ratio.

Distribution of Bacterial Taxa and Phylotypes.A total of 3,057,974 high-quality bacterial V4–V5Illumina sequences and 3856 OTUs were obtained from the fifth samples after highthroughputsequencing of the 16S rRNA gene, almost all these reads (99.96%) had the lengths of 300–450 bp, and average read length is 414 bp. The sequences numbers were differ among each sample, ranged from 58,942 to 70,041. Their coverage value was adequate, ranging from 98.9 to 99.1% (Table 2), with the number of OTUs increasing sharply before reaching a plateau; this indicated that at this evolutionary distance, the sequence number of each sample can well characterize the bacterial community of the sample (Fig. 1).The dominant bacterial phyla were *Acidobacteria* (34.32–44.09%), *Proteobacteria* (27.39–30.62%), *Actinobacteria* (7.61–10.94%), *Chloroflexi* (7.53–11.33%), *Planctomycetes* (3.36–5.21%), *Verrucomicrobia* (2.90–4.33%), *Bacteroidetes* (0.78–1.44%), *Gemmatimonadetes* (0.67–1.49%), *Firmicutes* (0.48–0.93%), and *Armatimonadetes* (0.41–0.74%)(Fig. 2). Together, they take possession of over 95% of the bacterial sequences from per mixed ratio forest types.

**Table 2:** Soil bacteria richness and diversity estimation under five mixing ratios of bamboo–broad-leaved forest.

Forest	Sample ID	Number of OTUs	ACE Index	Shannon’s Index	Coverage (%)
	A11	2,342	2,763.96	6.0453	99.10
A1	A12	2,412	2,883.25	6.0975	99.03
	A13	2,395	2,801.70	6.0229	99.09
	**Average**	**2383**	**2816.30**	**6.06**	**99.07**
	B11	2,458	2,846.60	6.3482	98.94
B1	B12	2,392	2,932.63	5.9669	98.98
	B13	2,327	2,817.89	6.075	98.91
	**Average**	**2,392**	**2,865.70**	**6.13**	**98.94**
	C11	2,285	2,761.33	5.8007	99.04
C1	C12	2,177	2,697.71	5.6904	98.98
	C13	2,237	2,743.12	5.7691	98.98
	**Average**	**2,233**	**2,734.05**	**5.75**	**99.00**
	D11	2,423	2,906.25	6.0741	98.98
D1	D12	2,387	2,826.78	6.0304	99.07
	D13	2,429	2,825.72	6.2019	99.09
	**Average**	**2,413**	**2,852.92**	**6.10**	**99.05**
	E11	2,719	3,223.74	6.1895	98.88
E1	E12	2,226	2,712.04	5.8366	99.06
	E13	2,178	2,626.24	5.8907	99.11
	**Average**	**2,374**	**2,854.01**	**5.97**	**99.02**

Bacterial α-Diversity and Differences inCommunity Structure.In accordance with OTU alpha diversity estimated by the ACE index,the highest bacterial richness occurred in the B1 site soils (mean 2,865.70, respectively) followed byE1, D1, and A1 (average 2,854.01, 2,852.92 and 2816.30, respectively), whereas C1 showed the lowestbacterial richness (average 2,734.05; Table 2)). Bacterial diversity, in terms of the Shannon index, was highest in B1 and D1 soils (mean 6.13 and 6.10, respectively) followed by E1, A1 (mean 5.97 and 6.06, respectively), and it was the lowest in C1 site soil (mean 5.75; Table 2). It can be found that a mixing ratio will affect soil bacterial diversity to some extent from above results.

Each soil bacterial at the bacterial phyla level shown a distinct distribution among the five mixing ratios forest (Fig. 2). It is worth noting that the relative abundance of soil dominant bacterial in C1 site is significantly different from the soil of the other four mixed ratio forest sites. The *Acidobacteria* were least abundant in C1 (44.09%) followed by E1 forest (40.34%), A1 (38.58%), D1 (36.56%), and B1 forest (34.32%), while *Acidobacteria* increased in relative abundance from A1 to C1 forests and decreased from C1 to E1 forests. Conversely, the *Proteobacteria* were less abundant in B1 (27.39%) and C1 (29.16%) sites than in the A1 (29.54%), E1 (30.42%) and D1 sites (30.63%). Generally, the trend in taxadominant relative abundance was decreasing from A1 to C1 but increasing from C1 to E1.

### Impact of mixed forest ratio-associated environmental factors on bacterial community composition and diversity

**Figure 3:**
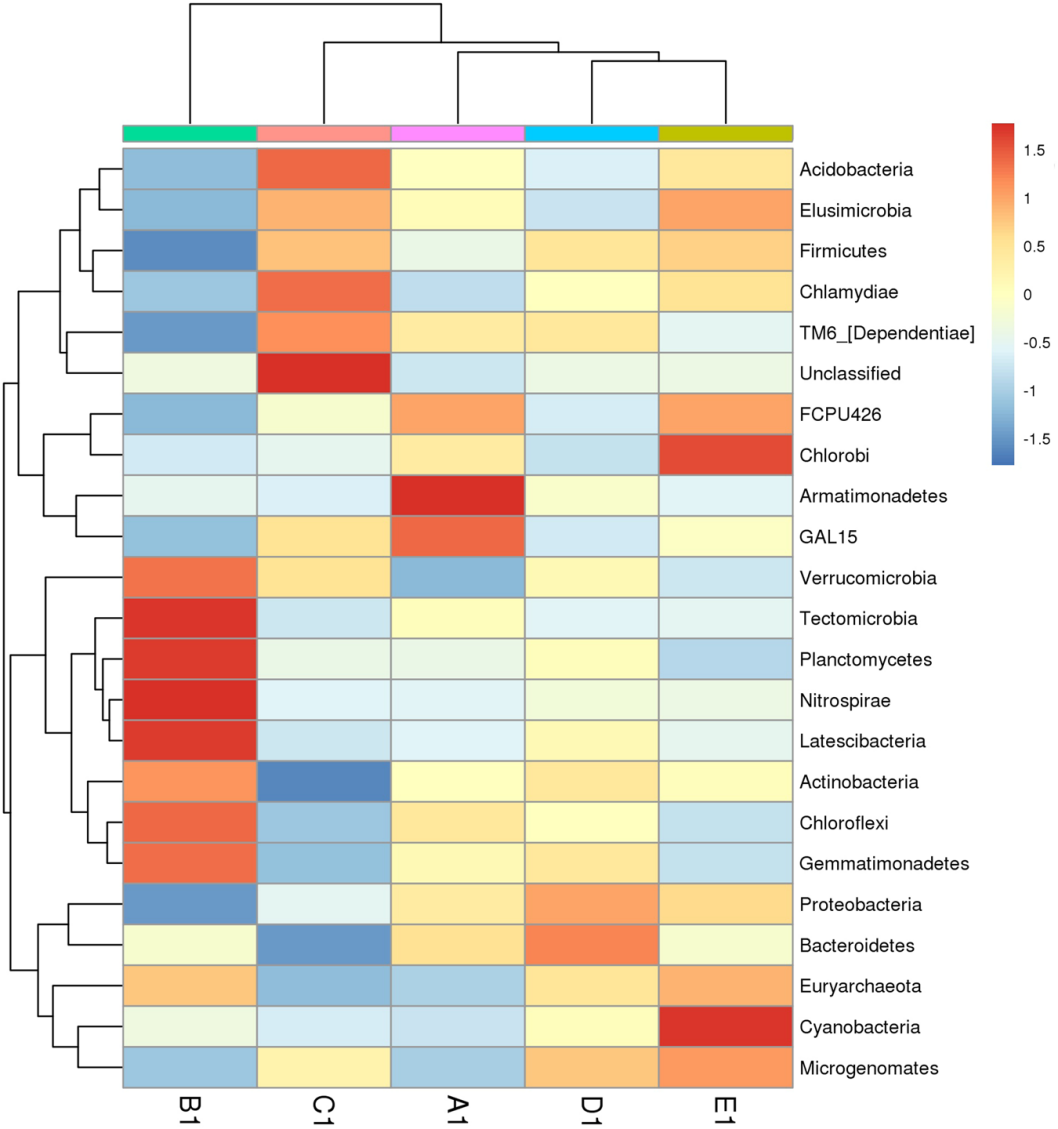
Heatmap diagram shown the dominant 23 bacterial OTUs under five mixing ratios of bamboo–broad-leaved forest.

**Figure 4:**
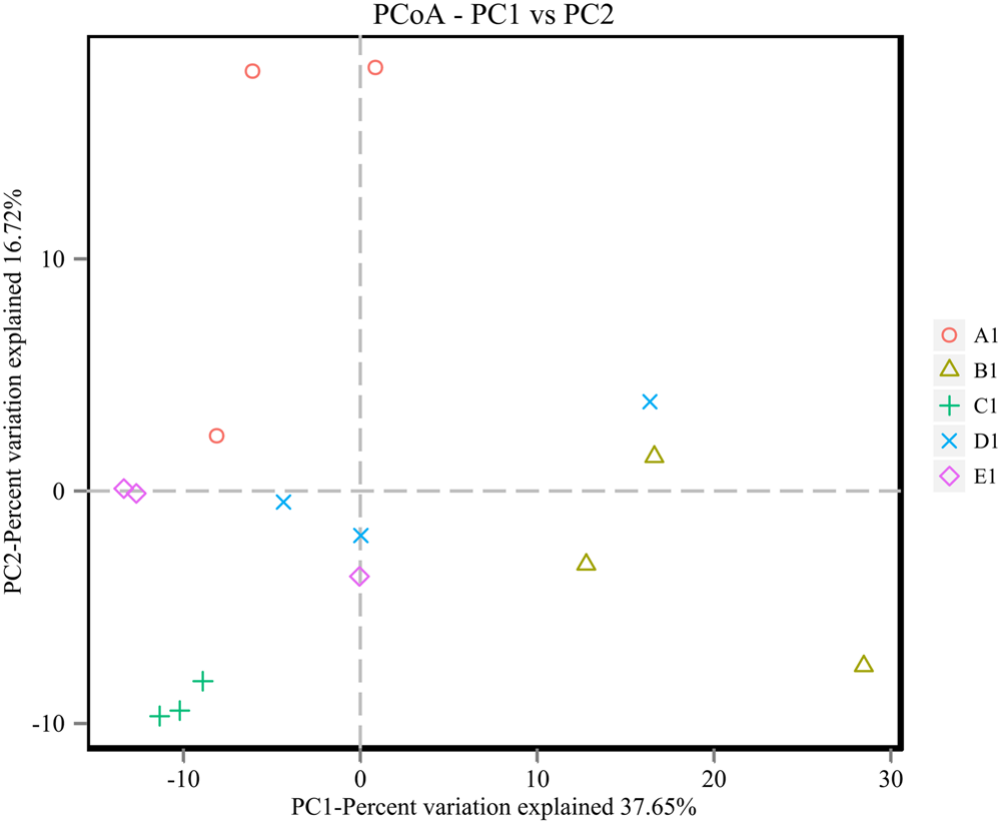
PCoA plot of soil bacterial communities from five mixing ratio types forests based on the Bray-Curtis distance.

To examine soil bacterial compositionat the phyla level, a heatmap analysis of the 23 richest OTUs (based on the bacterial community profiles) was used, which highlighted their relativedistributions and abundances across the sampled forest sites. As Fig. 3 shows, the composition of soil bacterial community structure were differed among the five mixing ratio types. The D1 and E1 groups clustered, and were separated from the A1 and C1 groups, suggesting a marked difference in the microbial community structure between B1 forests and other four mixed types.The same result can be found from Principal Coordinates Analysis (PCoA) of soil bacterial communities from the five mixing ratio types forest based on the Bray-Curtis distance from the perspective of species diversity(Fig. 4). In general, the two PCoA account for 54.37% of the differences between different communities. The PCoA score plot shows the D1 and E1 soils with their characteristic bacterial communities, in which C1 soil samples were well separated from the others, whereas there was little relation between D1 and E1 samples. These results indicated that the mixed ratio had the highest impact on soil bacterial communities took to support mixed bamboo and broad-leaved forest.

**Figure 5:**
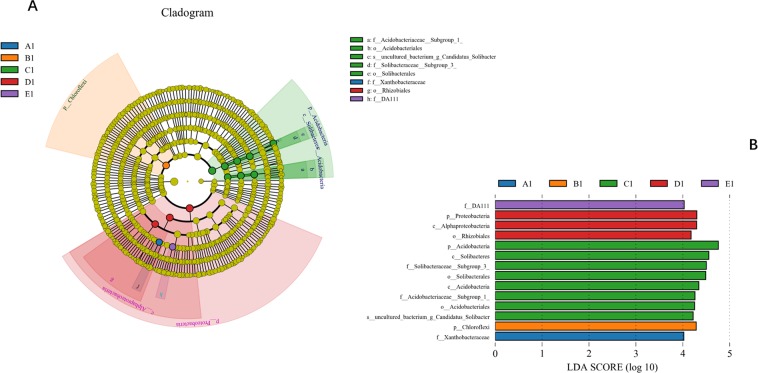
Thestatistically significant biomarkers among five mixing ratios of bamboo–broad-leaved forests analyzed by LEfSe. (**A**) Soil bacterial gene that were differentially among five mixing ratios of bamboo–broad-leaved forests cladogram generated from LEfSe analysis. (**B**) Differences in key OTUs identified as differentiating among five mixing ratios of bamboo–broad-leaved forests.

Furthermore, our study also used LEfSe (LDA effect size) to analyze the five forest sites to find statistically significant biomarkers among them. As shown by its histogram (Fig. 5), eight bacteria in C1 soils that are statistically significantly different from the other sites, and there are also consistent differences in biology. The predominant bacteria in the C1 site belonged to the phylum capable of decomposing soil organic materials (*Acidobacteria*), and the C1 site overrepresented classes including *Solibacteres* and *Acidobacteria* (found exclusively in C1 samples). At the order level, the bacterial taxa in the C1 site wasoverrepresented by*Solibacterales* and *Acidobacteriales*.

**Figure 6:**
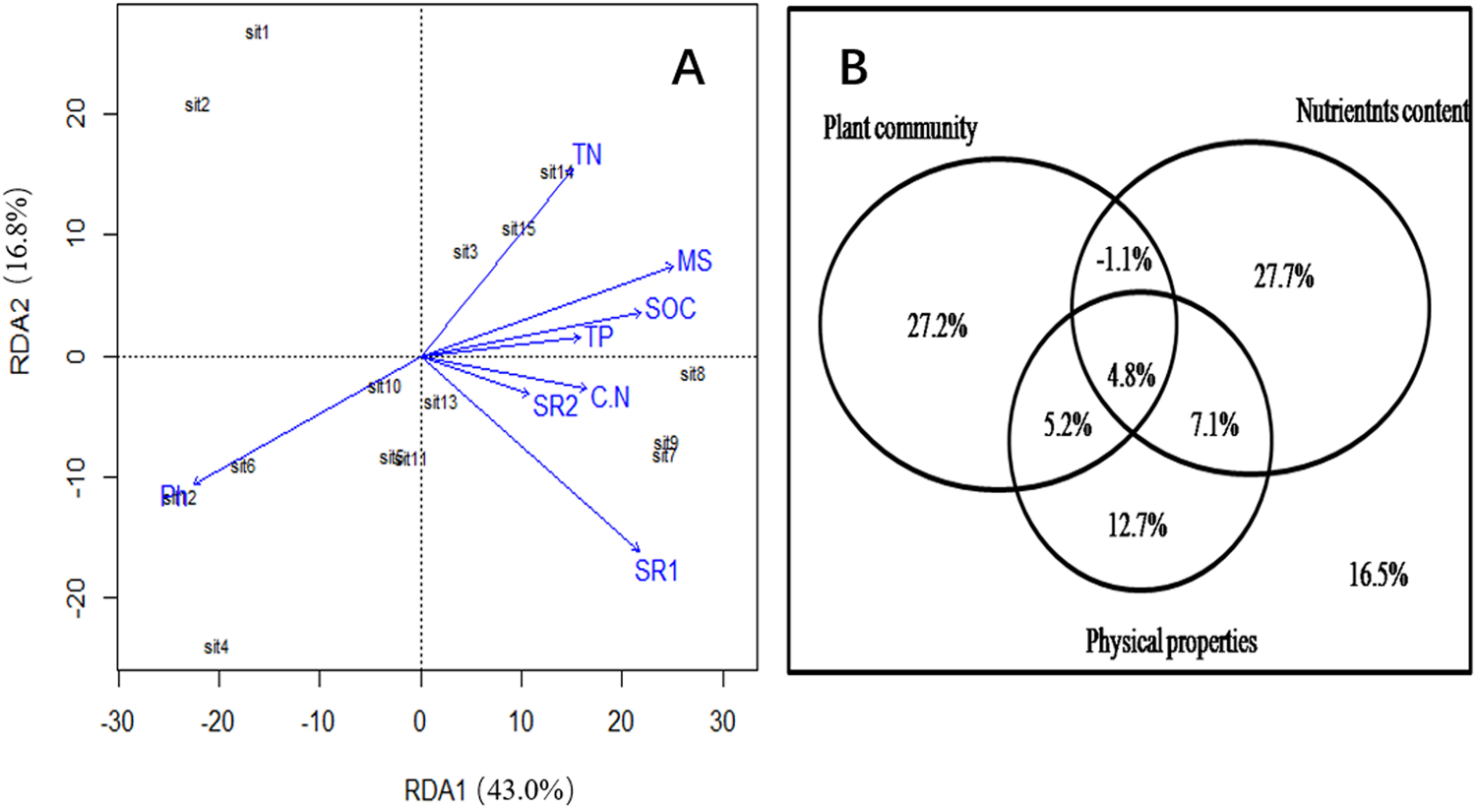
(**A**) RDA analysis showing the correlations between microbial community structure and environmental factors. (**B**) Venn diagram used to interpret the influence ofenvironmental factors on soil microbial communities. Environmentalfactors as shown in Table 1.

According to the RDA analysis and a Monte Carlo permutation test, the eight environmental parameters (Table 1) accounted for 83.5% of the variation in the observed bacterial community structure. Axis 1 explained 43.0% of this variation and axis 2 explained another 16.8% (Fig. 6A). The variation partition analysis (Fig. 6B) indicated that two plant community factors, two of soil physical properties, and four corresponding to soil nutrient content explained 27.2%, 12.7%, and 27.7% of bacterial community variation, respectively. Considered individually, the major factor that explained most of the variation in soil bacterial community composition was the SR1 of the plant community (*R*^2^ = 0.7073, *p* = 0.001), followed by MS (*R*^2^ = 0.5740, *p* = 0.014), pH (*R*^2^ = 0.5468, *p* = 0.007), TN (*R*^2^ = 0.4922, *p* = 0.016), and SOC (*R*^2^ = 0.4006, *p* = 0.044).

## Discussion

Our previous research results have shown that the mixed ratio has a certain effect on the inter-species relations in bamboo and broad-leaved mixed forests^28^. To some extent, the presence of additional broad-leaved trees can improve the soil environment and therefore provide better soil water and soil nutrient supplies for the affiliated bamboo plants. This is because the broad-leaved forest stands have higher soil respiration rate than other forest types^29^. For example, the soil moisture content increases as the broadleaf tree expands to the coniferous tree forest. As such, the broad-leaved forest on the upslope has a positive impact on the bamboo forest on its lower, slope-situated bamboo. In the current study, the moso bamboo growing in 20–30% broad-leaved forest had a high plant species richness in the tree layer; hence, by having more tree species, especially different ones such as conifers and broad-leaved plants, the greater the differences in their leaf and litter chemistry modify soil physicochemical properties^9^. It is known that as forest stand structure changes, the amount of light available differs; this can directly affect the composition of understory plants^30–32^. This may explain why soil physicochemical properties were higher in bamboo and broad-leaved mixed forests with a mixing ratio of 20–30% than in the other forest types. We also found that soil nutrient elements had higher values in bamboo and broad-leaved mixed forest with a mixing ratio of 20–30% (Table 1). The broad-leaved forest component in moso bamboo stands should have an optimal proportion. This is because although a higher proportion of broad- leaved forest in moso bamboo forests will generally affect the growth of moso bamboo, if this proportion is too high, the broad-leaved trees become the main species, and the large canopy formed by their crowns will adversely affect illumination conditions for bamboo.

Using high throughput sequencing of the 16S rRNA gene, we assessed bacterial community composition and diversity in soils of five mixing ratios of bamboo–broad-leaved mixed forest collected from Yongan Fujian, China. In this study, the ACE and Shannon indexes revealed differences in the complexity of soil microbial communities across the five forest types, namely a richer bacterial community in moso bamboo with 10–20% broad-leaved forest than in other mixed-ratio forest; the lowest value was in moso bamboo with 20–30% broad-leaved forest. Lin^9^ found the bamboo invasion mainly increases the bacterial diversity of the bamboo-associated soil community. Wang^32^ also found the moso bamboo forest had higher soil bacterial diversity than bamboo and broad-leaved mixed forest or pure broad-leaved forest. Hence, the understory plants in our five forest types were largely dissimilar. In addition, the collection of bamboo shoots and harvestable bamboo timber can vary greatly across the five forest sites we studied; thus, our results could have been further influenced by different moso bamboo management practices that disturb surface soils, which could have contributed to increased soil bacterial diversity^9,33,34^. The clear differentiation in the PCoA mapping indicated that the bacteria in the C1 stand were well separated from other samples, with some correlation between D1 and E1. These results point to the mixing ratio—that is, the ratio of broad-leaved tree crown projection over the plot area—in the bamboo-wide mixed forest playing a crucial role in the formation of microbial communities.

Our LEfSe analysis showed that *Acidobacteria* (*Solibacteria*,*Solibacteriales*,*Acidobacteriales*) under the 20–30% mixing ratiodiffered significantly in abundance when compared with other forests. Many studies have also found that *Acidobacteria* is widely distributed in agricultural land and forest soils^35–37^. Most studies suggest that *Acidobacteria* is an oligotrophic organism^38^ as well as a multi-purpose heterotrophic organism that has a slower metabolic rate under low nutrient conditions. In contrast, Han^39^ found that such acid bacteria could not be isolated from bamboo forest soil by using a dependent culture method. Therefore, further exploration of the functional role of *Acidobacteria* is warranted in bacterial communities of different soil mixtures under mixed bamboo and broad-leaved forest stands.

Previous studies showed that soil pH was closely correlated with bacterial diversity and soil microbial communities^40–42^. However, other work has found that soil water content is a main driver of soil microbial community structure rather than soil pH^43,44^. In our study, RDA was used to explore relationships between environmental factors and soil microbial community structure. These results confirmed that the soil bacterial community composition of mixed bamboo and broadleaved forest was negatively correlated with soil pH and soil water content, a finding consistent with other reports on terrestrial soil research^45,46^. Fierer and Jackon^47^ found evidence that soil pH was the best predictor of soil bacterial diversity in a range of terrestrial and aquatic environments. More recently, Xue^42^ demonstrated that different bacterial species have different adaptability to environmental pH. In addition, most bacteria have weak growth tolerance. Both are likely to explain the soil bacterial community structure’s significant correlation with soil pH. Changes in soil water content not only cause changes in soil chemical processes but also other factors in the soil, thereby influencing the distribution of soil microbial communities.

In our study, total N and SOC were positively related to bacterial diversity and soil microbial communities, and a similar pattern has been found in many other studies conducted at various scales^42,48–51^. For example,Nielsen^52^ studied the response of soil microbial community distribution to vegetation type and soil physicochemical properties at the landscape scale, finding that changes in vegetation type have no direct influence upon soil microbial community structure but did influence soil nutrients. Moreover, a changed forest microenvironment may indirectly have certain effects on the distribution of soil microbial community structure^53,54^. Finally, it has also been shown that changes to soil microbial community structure across space can arise from the influence of varying vegetation growth states and forest biomass^55^. In terms of the plant community, soil microbial community structure was significantly correlated with SR1 in our study. The species richness of the arbor layer should cause discernable differences in the microenvironment within the forest, and there is great variation in leaf traits and leaf area indexes of co-occurring tree species. In the form of litter, these leaf differences can change soil physicochemical properties, and also have an important impact on soil microbial community and enzyme activities. This could explain why the soil microbial community differed among the five mixed-ratio sites of bamboo broad-leaved mixed forest. Therefore, the dominant-associated tree species in the mixed forest of bamboo and broad-leaved forest should exert the greatest impact on its soil microbial community distribution. In a follow-up study, we plan to further explore the effects of different broad-leaved species on soil microbial community structure and diversity.

## Conclusions

Our research indicates that the microbial community structure and diversity of the soil in the bamboo and broad-leaved mixed forests are different as the mixing ratio is changed in a stand. The mixed ratio (as represented by the width and size of the broad-leaved tree crown over the plot area) affected both soil characteristics and the microbial community. A differing ratio not only causes species diversity to change in the forest, which is accompanied by different litterfall yields, but it also underpins differing physical and chemical properties of the soil. There is a correlation between bacterial community and soil and vegetation factors under different mixing ratios. In the stand with a mixed ratio of 10–20%, the bacterial diversity index is higher; however, the diversity was lowest in the 20–30% stands. Among the 20–30% forest soil, *Acidobacteria* (*Solibacteria*, *Solibacteriales*, *Acidobacteriales*) was more abundant than in soils from other mixed-ratio stands. Changes in microbial communities were associated with species diversity in tree layers, availability of soil nutrients (SOC and TN), and changes in soil physical properties (MS, pH).Mechanistically understanding how the mixing ratio of broad-leaved trees species’ crowns in bamboo production areas affects microbial community is a promising research avenue.

## Materials and methods

### Study area description

The mixed bamboo and broad-leaved forests area is located in China’s Tianbaoyan Nature Reserve (117°28′03″E—117°35′28″E, 117°28′03″E—117°35′28″E), which lies near the junction of Xiyang, Shangping, and Qingshui Townships (towns) in the eastern part of Yong’an City (Fujian Province). This protected area spans middle-elevation and low-elevation mountainous landforms, the remaining clouds of Daiyun Mountain, at 580–1604.8 m a.s.l., where the main type of soil is red earth. The climate is a medium subtropical southeast monsoon type: annual average temperature is 15 °C, minimum temperature reached –11 °C and maximum temperature reached 40 °C, with an annual average relative humidity >80% and an average frost-free period of ca. 290 days per year^56^. The land coverage of bamboo forest in the protected area is 96.8%, and is mainly distributed below 800 m a.s.l., with the main bamboo species being moso bamboo. The primary tree species associated with moso bamboo are *Castanopsiscarlesii*,*Castanopsisfargesii*,*Alniphyllumfortunei*,*Cinnamomumcamphora*, *Liquidambar formosana*,and *Sassafras tzumu*, as well as*Phoebe zhennan*,*Schimasuperba*,*Quercuschenii*,*Myricarubra*,and *Choerospondias* spp.

### Filed sites and soil sample collection

Field experiments were set up in November 2017, based on the site conditions consistent with the overall exploration of mixed forests in the protected area. A selection of mixed forests containing bamboo and broad-leaved trees growing under the same site conditions were used. Across their distribution areas, typical sampling methods were used to set up five sites with different mixing ratios of tree to bamboo. Specifically, the sampling plots for the mixed proportions were divided according to the percentage of summed projected area of live broadleaf tree crowns: 10% or less(A1), 10–20%(B1), 20–30%(C1), 30–40%(D1), and more than 40%(E1). By manually measuring the things of the broad-leaved trees in the stand, the crown width in the north-south direction, the average of the two is calculated, and the average value is the broad-leaved tree crown. The main broad-leaved species in the five mixed ratio plots were *Castanopsisfargesii*,*Schimasuperba*,*Alnus japonica*,*Choerospondiasaxillaris*, *Sassafras tzumu*,*Schimasuperba*,and *Liquidambar formosana*, and the broad-leaved tree species in the forest are the same. Each plot was 20 m × 20 m in size, and a total of 15 plots were established, three per forest ratio class, the minimum distance between plots was 500 mto avoid pseudo replication. Soil samples were collected from the surface (0–10 cm depth) of each plot with a soil auger (2.5 cm in diameter). To do this, eight soil points were selected (following the S-type sampling method) from per plot, and their samples mixed to form one composite replicate sample per plot. Once collected, after quartering, all soil samples were immediately divided into two portions, with one portion quickly refrigerated and taken to the laboratory for storage at –80 °C for subsequent DNA analysis. The other portion was air dried at the laboratory, and its impurities were removed (e.g., largepieces of plant material, gravel, earthworms) and then the sample was ground. Non-refrigerated samples were crushed, sifted, and sealed in bags for later determination of soil nutrient content and enzyme activity.

### Vegetation study

Species richness (SR) of the plant communities in the plots was measured using the quadrat method. We identified all shrubs and herbaceousplants, and measured their basal diameter, height, andcrown width or degree of crown closure^57^. Weestimated species richness at each site based only on thespecies that occurred inside the 5 × 5 m^2^ plots for trees and shrubs and inside the 1 × 1 m^2^ quadrates for herbaceous species^57^. The botanical nomenclature follows theInstitute of Botany, Academia Sinica^57,58^. Species richness, which represents the transformed number of species recorded in a sampling area^59^, the indices are calculated as follows

Species richness index = (S – 1)/ln N; where S is the number of species and N is the total number ofindividuals

### Soil physicochemical properties

Soil pH was determined using an electrode pH meter (Sartorius PB-10, Germany) after shaking the soil water liquid suspension (1:5 wt/vol)^31,60^. Soil moisture (MS) was analyzed by weighing the soil sample and calculating the mass lost after oven-drying it at 105 °C to a constant weight (ca. 24 h)^42^. Soil total carbon (SOC) was then determined using an elemental analyzer (Elementar, Germany) and total nitrogen (TN) content was assessed via the Kjeldahl method^20,31^. Total phosphorus (TP) was measured by spectrophotometry after wet digestion with HClO_4_-H_2_SO_4_^31,61^.

### DNA extraction

Total bacterial DNA was extracted from each soil sample using the Power Soil DNA Isolation Kit (MO BIO Laboratories), according to the manufacturer’s protocol. DNA quality and quantity were assessed by the ratios of 260 nm/280 nm and 260 nm/230 nm, respectively. Then, the DNA was stored at −80 °C until further processing^34^.

### PCR amplification

#### rRNA gene

The V3-V4 region of the bacterial 16S rRNA gene was amplified with its widely used primer pair (forward primer, 5′-ACTCCTACGGGAGGCAGCA-3′; reverse, primer, 5′-GGACTACHVGGGTWTCTAAT-3′) and combined with adapter sequences and barcode sequences^62,63^. PCR amplifications were performed in a total volume of 50 μl, which contained 25 μl of bμffer(KOD FX Neo Buf(2×)), 1 μl of KOD FX Neo(TOYOBO) DNA Polymerase, 10 μl of 2Mm dNTP, 1.5 μl of 10 μM of each primer, ~60 ng of genome DNA, andddH2O to bring the mixture to volume. Thermal cycling conditions were as follows: an initial denaturation at 95 °C for 5 min, followed by 15 cycles at 95 °C for 1 min, 50 °C for 1 min, and 72 °C for 1 min, with a final extension at 72 °C for 7 min^62^. The PCR products from the first PCR round were purified through VAHTSTM DNA Clean Beads^62^. A second round PCR was performed in a 40-μl reaction that contained 20 μl of 2× Phμsion HF MM, 8 μl of ddH_2_O, 2 μl of each primer (10 μM), and 10 μl of the PCR product (s) from the first round. Thermal cycling conditions were as follows: an initial denaturation at 98 °C for 30 s, followed by 10 cycles at 98 °C for 10 s, 65 °C for 30 s and 72 °C for 30 s, with a final extension at 72 °C for 5 min^62^.

#### IlluminaMiSeq

Finally, all PCR products were quantified with the Quant-iT™ dsDNA HS Reagent and pooled together^62^. High-throughput sequencing analysis of bacterial rRNA genes was performed on the purified, pooled sample using the IlluminaHiSeq. 2500 platform (2 × 250 paired ends) at Biomarker Technologies Corporation, Beijing, China.

### Sequence preprocessing

According to the overlapping relationship between the PE reads, the double-ended sequence data obtained by HiSeq sequencing was spliced into a sequence of tags, and the quality of reads and the effect of merging were quality-controlled and filtered. The tree main steps were as follows: 1) PE reads splicing: FLASH v.1.2.7 software was used, according to the minimum overlap length of 10 bp and the maximum allowable mismatch ratio of overlap area of 0.2, the reads of each sample were spliced, and the resulting splicing sequence was the raw tags data; 2) Tag filtering: Trimmomatic v.0.33 software filtered the spliced raw tags to obtain high-quality tags data; and 3) Removal of chimeras: this was done using UchiME v.4.2 software, which identified and excised chimeric sequences to yield the final valid data set. By using UCLUST^64^ in QIIME (v.1.8.0) software^65^, the clusters were grouped at the 97% similarity level, and each corresponding OTU was obtained. Every OTU was then classified according to the Silva taxonomy database.

### Data analysis

Statistical analysis of the ACE and Shannon indexes was performed using Mothur (v.1.30) software^66^. One-way analysis of variance (ANOVA) was used to compare differences in means of the soil physicochemical properties, relative microbial content, and α-diversity index across the five mixed-ratio forest types. The Duncan test was further employed to test for significant differences at the P = 0.05 level^44^. According to the species composition and relative abundance of each sample, the heatmap were performed based on the Rpackage “pheatmap” to extract the species at phyla level. Redundancy analysis (RDA) and partial RDA analysis were performed with the R package “vegan”,Forward selection wasbased on Monte Carlo permutation tests (permutations D 999)^67^.

## Data Availability

The datasets generated during the current study are not publicly available due to the data also forms part of an ongoing study but are available from the corresponding author on reasonable request.
